# Simulation of Frost-Heave Failure of Air-Entrained Concrete Based on Thermal–Hydraulic–Mechanical Coupling Model

**DOI:** 10.3390/ma17153727

**Published:** 2024-07-27

**Authors:** Xinmiao Wang, Feng Xue, Xin Gu, Xiaozhou Xia

**Affiliations:** College of Mechanics and Engineering Science, Hohai University, Nanjing 211100, China; 211308010040@hhu.edu.cn (X.W.); xuefeng12@hhu.edu.cn (F.X.);

**Keywords:** air-entrained concrete, effective thermal conductivity, effective permeability coefficient, fracture phase-field method, thermal–hydraulic–mechanical coupling

## Abstract

The internal pore structural characteristics and microbubble distribution features of concrete have a significant impact on its frost resistance, but their size is relatively small compared to aggregates, making them difficult to visually represent in the mesoscopic numerical model of concrete. Therefore, based on the ice-crystal phase transition mechanism of pore water and the theory of fine-scale inclusions, this paper establishes an estimation model for effective thermal conductivity and permeability coefficients that can reflect the distribution characteristics of the internal pore size and the content of microbubbles in porous media and explores the evolution mechanism of effective thermal conductivity and permeability coefficients during the freezing process. The segmented Gaussian integration method is adopted for the calculation of integrals involving pore size distribution curves. In addition, based on the concept that the fracture phase represents continuous damage, a switching model for the permeability coefficient is proposed to address the fundamental impact of frost cracking on permeability. Finally, the proposed estimation models for thermal conductivity and permeability are applied to the cement mortar and the interface transition zone (ITZ), and a thermal–hydraulic–mechanical coupling finite element model of concrete specimens at the mesoscale based on the fracture phase-field method is established. After that, the frost-cracking mechanism in ordinary concrete samples during the freezing process is explored, as well as the mechanism of microbubbles in relieving pore pressure and the adverse effect of accelerated cooling on frost cracking. The results show that the cracks first occurred near the aggregate on the concrete sample surface and then extended inward along the interface transition zone, which is consistent with the frost-cracking scenario of concrete structures in cold regions.

## 1. Introduction

Frost-heave damage is a prevalent issue for concrete structures in high and cold regions. Consequently, studying the frost resistance performance of concrete is crucial for enhancing the service life of concrete structures and ensuring their normal function. To improve the frost resistance of concrete, it is necessary to add an air-entraining agent to produce a large number of microbubbles when pouring concrete, with the size of these microbubbles ranging from tens of micrometers to hundreds of micrometers. Under the action of pore pressure, the unfrozen water in concrete flows into microbubbles through infiltration or capillary pores, releasing a certain degree of pore pressure and alleviating the internal stress of concrete. Powers [1] posited that the volume expansion of water in the pores during freezing compresses the unfrozen water, leading to an increase in internal stress in the concrete. The microbubbles in concrete play a storage role in the discharge of capillary water during the freezing process, thereby alleviating ice-crystal pressure. This model effectively explains the influence of microbubbles generated by air entrainment on pore pressure but overlooks the impact of matrix shrinkage caused by the suction effects of microbubbles during freezing. Scherer et al. [2,3] proposed a crystallization-pressure model where, during the cooling process, microbubbles within the cementitious material function as cryo-suction sites. This cryo-suction causes continuous shrinkage of the matrix, which offsets some of the pressure exerted by ice crystals as water freezes. The microbubbles in the matrix pump liquid water from the capillary pores into the microbubbles under the influence of cryo-suction. When microbubbles are filled with pore water, they lose their capability to store and extract water at low temperatures, leading to the redistribution of pore water around microbubbles. Mayercsik et al. [4] studied the redistribution of water inside these pores and calculated the volume of water discharged into microbubbles during cooling. The cooling of concrete materials proceeds from the surface to the inside, so there will be obvious temperature gradients and pore pressure gradients inside the concrete. Gong [5] studied the fluid flow patterns in cement-based materials with different pore pressures, temperatures, and entrained air contents. The results showed that the hydraulic flow depends on the microbubble content and pore pressure gradients, while the low-temperature suction flow depends on the temperature gradients and permeability. However, the calculation model used only focused on the distribution of single-size microbubbles. Wei et al. [6] studied the pore pressure distribution of saturated mortar under rapid cooling conditions, and a mathematical model was established for quantitatively simulating the temperature distribution of specimens. This model intuitively reveals the influence of concrete size on the temperature hysteresis effect. In addition, during the freezing period, the permeability of the cementitious material decreases as it is sucked and contracted, resulting in an uneven distribution of pore pressure and local stress around the microbubbles.

The frost-heave cracking of air-entrained concrete is a complex physical process involving water transfer, heat transfer, and the phase transformation of pore water as the temperature continuously decreases. This process is accompanied by dynamic changes in porosity and pore pressure. When the pore pressure reaches the limit of the matrix (including the internal ITZ), the material undergoes frost-heave cracking. To investigate the relieving effect of microbubbles on ice-crystal pressure, Dong et al. [7] established a finite element model of the thermal–hydraulic–mechanical coupling of cement-based materials at the microscale. In this model, the ice-crystal mechanism of pore water is considered: when the microbubbles are filled with water, the permeability coefficient of the matrix near the microbubbles becomes very small, creating an impermeable condition at the microbubble boundary. Based on the established model, the influence of microbubble content, size, and spacing on ice-crystal pressure was studied. However, due to the small size of the sample, the time scale must be very small; otherwise, it is difficult to capture the gradient changes in temperature and pressure. Therefore, Zhou et al. [8] established a fully coupled thermal–hydraulic finite element model of concrete that can reflect the water–ice phase transformation at the mesoscale. They explored the effects of various factors, including the permeability of the cement mortar and the ITZ, the thermal expansion coefficient of the ITZ, the aggregate volume fraction, and aggregate grading on the damage to concrete under freeze–thaw conditions. Xie et al. [9] reconstructed a three-dimensional mesoscale geometric model of concrete containing aggregates, mortar, an ITZ, and pores based on CT scanning. They utilized COMSOL Multiphysics software to analyze the effects of freeze–thaw cycles, the water–cement ratio, and air content on the mechanical properties of concrete under the combined influence of seepage, heat conduction, and structural deformation. Kenny Ng et al. [10] studied the freeze–thaw damage to the internal microstructure of cement paste caused by ice crystallization pressure. They used three-dimensional cohesive zone modeling techniques to simulate crack propagation in the porous system of cement paste under freeze–thaw conditions. The model was validated by comparing it with the microstructural characteristics of cement paste observed through scanning electron microscopy imaging techniques. Marcin Koniorczyk et al. [11] proposed a new thermo-hydro-mechanical coupling model that employs a non-equilibrium approach to simulate the phase-change kinetic equation of water freezing during freeze–thaw cycles in concrete. This equation was numerically solved using isotropic delayed damage theory, the finite element method, and the difference method. The model’s effectiveness was validated through comparison with experimental results, and it was applied to analyze freeze–thaw damage in saturated concrete walls. Olivier Coussy et al. [12] studied the freezing process of pore water in cement-based materials using the theory of unsaturated elasticity and estimated the critical spacing factor. They investigated the influence of pore size distribution on the critical spacing factor and internal pressure of cement-based materials through numerical simulation. During the freezing process, microbubbles generated by air entrainment and the phase change of pore water significantly impact the physical properties of the cement slurry, such as heat conductivity, permeability, and mechanical properties. This is crucial for designing air-entrained concrete and directly affects its frost resistance. However, directly meshing microscale structures like microbubbles and pore water at the microscale results in an excessively large computational model and makes calculations difficult. For this reason, it is also necessary to perform homogenization estimation on the air-entrained cement mortar to achieve a physical property characterization of the cement mortar that can reflect the distribution characteristics of microbubbles and the degree of the pore-water phase change.

For the estimation of the effective modulus, theoretical methods have tended to be perfect. The main methods widely used in concrete homogenization models include the self-consistent method [13], the Mori–Tanaka method [14], and the differential method [15]. The self-consistent method is implicit, and for composite materials with high-density inclusions, the generalized self-consistent method [16] is required. The Mori–Tanaka method is an explicit estimation method suitable for low-density inclusions. The differential method is a hierarchical estimation method, which forms a new matrix by adding some inclusions to the original matrix and then adds other inclusions to the base of the new matrix to form an updated matrix. This mode helps to solve the estimation problem of high-density inclusions. Therefore, for high-density inclusion materials such as concrete, the best strategy is to use a combination of the differential method and Mori–Tanaka method: that is, use cement mortar without microbubbles and pore water as the matrix, use microbubbles and pore water as the inclusions, and use the Mori–Tanaka method to estimate the effective modulus of the cement mortar; then, use the cement mortar as the matrix, use aggregates as the inclusions, and continue to use the Mori–Tanaka method to estimate the effective modulus of concrete. For the estimation of physical properties such as thermal conductivity and permeability, the volume average method is usually used [17], which is a weighted average estimation of thermal conductivity and permeability based on the volume proportion of each component.

Tracing and simulating the concrete fracture process is an important means of studying the frost resistance of concrete in cold regions. With frost cracking, the concrete surface is prone to peeling, and the location of frost damage is not known in advance. Therefore, the cohesive element method depending on FEM mesh [18,19] and the extended finite element method requiring preset cracks [20,21,22] are not suitable. For numerical methods based on traditional damage mechanics [23,24,25], the cracking effect exerted by damage is not very good, especially for homogeneous materials, due to the lack of special treatment for deformation localization. Bazant proposed a nonlocal damage architecture [26,27,28], which solved the problems of mesh sensitivity and size effects and achieved a good damage localization effect. However, stress locking cannot be avoided because of the irrational energy release mechanism. Recently, peridynamics [29,30,31,32,33,34,35,36,37] have become popular for naturally simulating the entire process of crack initiation and propagation; however, the computational cost is still very high, and there are significant boundary effects, making it difficult to simulate concrete surface cracking accurately. Another popular numerical method for simulating crack propagation is the phase-field method [38,39,40,41,42,43], which is a nonlocal gradient method for damage. It takes damage as a phase variable and establishes the differential equation of the phase field through energy functional variation [44,45,46,47,48]. Because there is no need to update the crack geometry, the phase-field approach has been successfully applied to the simulation of the corrosion-induced cracking of reinforced concrete [49], the shrinkage-induced cracking of early-age concrete, and the quenching fracture of ceramic plates [50]. However, the fracture phase-field method has not yet been applied to the simulation of the concrete frost-heave fracture under the thermal–hydraulic–mechanical coupling interaction.

In summary, current simulations of concrete frost cracking do not account for the effects of pore structure characteristics and microbubbles generated by air entrainment on the frost resistance of concrete. Therefore, this article first focuses on the estimation of the macro thermal and permeability properties of air-entrained mortar, providing an expression of average pressure with temperature and permeability considering the ice-crystal transition mechanism. Then, at the mesoscale, a finite element thermal–hydraulic–mechanical coupling model of concrete is established based on the fracture phase-field method. The established homogenization model for thermal conductivity and permeability coefficient is applied to the phases of cement mortar and the ITZ. Finally, the buffering effect of microbubbles on the concrete ice-crystal pressure and the adverse effect of accelerated cooling on frost cracking during freezing is explored.

The structure of this paper is as follows: Section 2 focuses on the homogenization of the thermal and permeability properties of cement mortar, reflecting the characteristics of the internal pore structure and microbubble content during freezing, and the average pore pressure with the cooling process is derived and discussed for concrete with different entrained air contents. Section 3 introduces the fracture phase-field method to be used. Following this, Section 4 establishes the thermal–hydraulic–mechanical coupling finite element model of concrete at the mesoscale, where the fracture phase-field method is introduced to simulate the frost-induced cracking process, and the established estimation model for thermal conductivity and the permeability coefficient is applied to the cement mortar and ITZ phases. In Section 5, the simulated frost damage based on the established coupling numerical model is presented, and the effect of microbubbles in relieving ice-crystal pressure and the adverse effect of accelerated cooling on frost cracking during freezing are explored. Relevant conclusions are summarized and discussed in Section 6.

## 2. Estimation of Effective Properties of Cement Mortar and Average Pore Pressure

In this section, based on homogenization theories and the ice-crystal transition mechanism of pore water, the evolution law of effective thermal conductivity and permeability considering the internal microstructure of cement mortar is explored, respectively. Then, the average pore pressure of the porous mixture is derived and discussed for concrete with different entrained air contents.

### 2.1. Effective Thermal Conductivity of Air-Entrained Mortar

Considering the change in porosity during the phase transition of pore water inside the cement mortar, the expression for the effective thermal conductivity $\lambda_{eff}$ of cement mortar at low temperatures (below zero degrees Celsius) is derived as follows:

(1) $$\lambda_{eff}=\Phi^{\prime }s_{w}\lambda_{w}+\Phi^{\prime }s_{c}\lambda_{c}+(1-\Phi^{\prime })\lambda_{m}$$

where $s_{w}$ is the pore-water saturation, $s_{c}$ is the pore-ice saturation, $s_{w}+s_{c}=1$, $\lambda_{w}$ is the thermal conductivity of pore water, $\lambda_{c}$ is the thermal conductivity of pore ice, $\lambda_{m}$ is the thermal conductivity of the matrix, and $\Phi^{\prime }$ is the porosity after phase transformation. Assuming that the mortar matrix is isotropic and its effective modulus and Poisson’s ratio are ***E*** and ν, respectively, according to the theory of microscopic inclusions, the expression for the porosity of the cement mortar $\Phi^{\prime }$ after the phase transformation can be obtained as the following expression:

(2) $$\Phi^{\prime }=\Phi_{0}(1+s_{c}\alpha^{E}\varepsilon_{v}^{*})$$

where $\Phi_{0}$ is the initial porosity, i.e., the porosity for no phase transformation of pore water, $\varepsilon_{v}^{*}$ is the Eigenstrain generated by the phase transformation of pore water turning into ice, and $\alpha^{E}$ is the bulk part of the Eshelby tensor ***S***. For capillary pore water, it can be regarded as a columnar fiber inclusion, and the corresponding components of the Eshelby tensor ***S*** are listed as follows:

(3) $$\left\{\begin{array}{c} S_{1111}=S_{2222}=\frac{5-4\nu }{8(1-\nu )}\\ S_{1122}=S_{2211}=\frac{-1+4\nu }{8(1-\nu )}\\ S_{1133}=S_{2233}=\frac{\nu }{2(1-\nu )}\\ S_{1212}=S_{2121}=\frac{3-4\nu }{8(1-\nu )}\\ S_{1313}=S_{3131}=S_{2323}=S_{3232}=\frac{1}{2}\\ other=0 \end{array}\right.$$

Assuming the intrinsic strain of the capillary water–ice-crystal process to be $\varepsilon_{11}^{*}=\varepsilon_{22}^{*}=\varepsilon_{33}^{*}=\frac{\varepsilon_{v}^{*}}{3}$, the actual normal strains generated are as follows:

(4) $$\left\{\begin{array}{c} \varepsilon_{11}=S_{1111}\varepsilon_{11}^{*}+S_{1122}\varepsilon_{22}^{*}+S_{1133}\varepsilon_{33}^{*}=\frac{5-4\nu }{8(1-\nu )}\varepsilon_{11}^{*}+\frac{4\nu -1}{8(1-\nu )}\varepsilon_{22}^{*}+\frac{\nu }{2(1-\nu )}\varepsilon_{33}^{*}\\ \varepsilon_{22}=S_{2211}\varepsilon_{11}^{*}+S_{2222}\varepsilon_{22}^{*}+S_{2233}\varepsilon_{33}^{*}=\frac{4\nu -1}{8(1-\nu )}\varepsilon_{11}^{*}+\frac{5-4\nu }{8(1-\nu )}\varepsilon_{22}^{*}+\frac{\nu }{2(1-\nu )}\varepsilon_{33}^{*}\\ \varepsilon_{33}=S_{3311}\varepsilon_{11}^{*}+S_{3322}\varepsilon_{22}^{*}+S_{3333}\varepsilon_{33}^{*}=0 \end{array}\right.$$

Then, the actual bulk strain can be obtained as

(5) $$\varepsilon_{v}=\varepsilon_{11}+\varepsilon_{22}+\varepsilon_{33}=\frac{1}{2(1-\nu )}(\varepsilon_{11}^{*}+\varepsilon_{22}^{*}+2\nu \varepsilon_{33}^{*})=\frac{1+\nu }{3(1-\nu )}\varepsilon_{v}^{*}=\alpha^{E}\varepsilon_{v}^{*}$$

where $\alpha^{E}=\frac{1+\nu }{3(1-\nu )}$, with ν being the effective Poisson’s ratio of the mortar matrix. It is assumed that the densities of water and ice at low temperatures do not change with temperature: that is, the density of water is always 1 g/cm^3^, and the density of ice is always 0.9 g/cm^3^. Therefore, it is easy to calculate the value of the Eigenstrain, i.e., $\varepsilon_{v}^{*}=\frac{1}{9}$.

For air-entrained mortar with an internal microbubble content of c, according to the Mori–Tanaka estimation formula, it is easy to obtain its effective modulus as

(6) $$\left\{\begin{array}{c} \bar{K}=\frac{4G\left(1-c\right)}{3c}\\ \bar{G}=G\left(1-c\right){\left(1+\frac{2c}{3}\right)}^{-1} \end{array}\right.$$

where *G* is the shear modulus of the mortar matrix. Then, the bulk part of the Eshelby tensor of air-entrained mortar is given by

(7) $$\alpha^{E}=\frac{3\bar{K}}{3\bar{K}+4\bar{G}}=\frac{1+\bar{\nu }}{3(1-\bar{\;\nu })}$$

Therefore, the thermal conductivity of the air-entrained mortar matrix ${\bar{\lambda }}_{m}$ can be obtained as

(8) $${\bar{\lambda }}_{m}=(1-c)\lambda_{m}+c\lambda_{a}$$

where $\lambda_{a}$ is the thermal conductivity of microbubbles. The effective thermal conductivity for air-entrained cement mortar can be estimated by

(9) $$\begin{array}{ll} \lambda_{eff} & =\Phi^{\prime }s_{w}\lambda_{w}+\Phi^{\prime }s_{c}\lambda_{c}+\left(1-\Phi^{\prime }\right){\bar{\lambda }}_{m}\leftarrow \\ & =\Phi^{\prime }s_{w}\lambda_{w}+\Phi^{\prime }s_{c}\lambda_{c}+(1-\Phi^{\prime })\{(1-c)\lambda_{m}+c\lambda_{a}\} \end{array}$$

**Figure 1:**
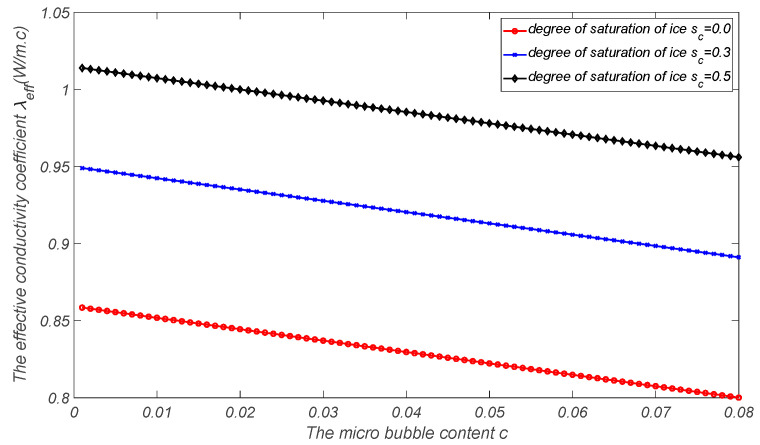
The effective thermal conductivity coefficient vs. microbubble occupancy for different degrees of saturation of ice.

**Figure 2:**
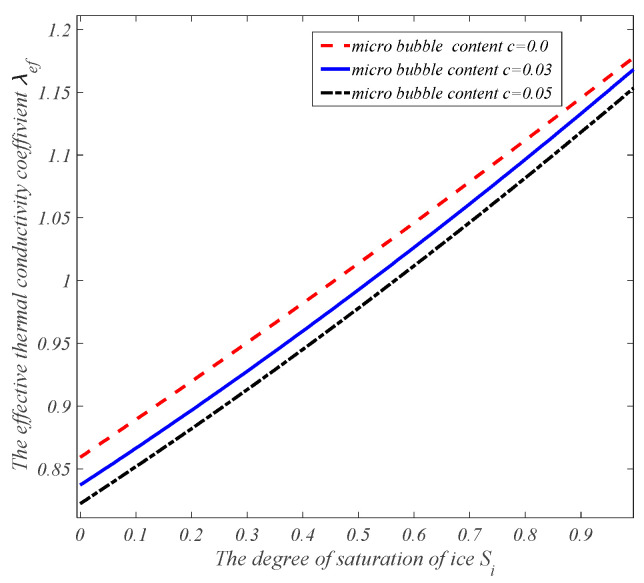
The effective thermal conductivity coefficient vs. the degree of saturation of ice for different microbubble occupancies.

By using the proposed estimation model of effective heat conduction, the heat conduction mechanism of air-entrained mortar during the freezing process can be explored. Assume that the thermal and mechanical parameters of each component in the air-entrained mortar are as follows: for the cement mortar matrix, the effective elastic modulus is E=20 GPa, Poisson’s ratio is ν=0.2, and the initial porosity is $\Phi_{0}=0.1861$. The thermal conductivity coefficient is $\lambda_{m}=0.93\;W/m\cdot K$. The thermal conductivity coefficients of microbubbles, capillary pore water, and capillary pore ice are $\lambda_{a}=0.025\;W/m\cdot K$, $\lambda_{w}=0.6\;W/m\cdot K$, and $\lambda_{c}=2.22\;W/m\cdot K$, respectively. The effective heat conduction curves of air-entrained cement mortar with the air content and degree of freezing can be obtained as shown in Figure 1 and Figure 2, where the thermal conductivity coefficient decreases with the increase in microbubble content and increases with the increase in the icing amount.

Embedding the above model for estimating the effective thermal conductivity of mortar into the multi-physical-field simulation of concrete materials or structures in a cold region can reflect the evolution of the effective thermal conductivity of cement mortar with the pore-water phase change in real time under the freezing and thawing regimes, and if the multi-physical-field coupled simulation of the freezing and thawing processes of concrete is carried out at a fine scale, the concrete matrix is replaced by the mortar matrix, and the effective thermal conductivity of the mortar matrix is estimated.

### 2.2. Effective Permeability Coefficient of Air-Entrained Mortar

Assuming that the pore water in the mortar is saturated and considering that the surface tension of capillary pores increases with decreasing pore size during the freezing process, the larger pore water in the mortar freezes first and then flows into the smaller pore water. The pore structure of cement mortar is divided into gel pores, capillary pores, and non-capillary pores. Assuming that their tortuosity and length are the same, the ratio of the number of pores with two different apertures under the same porosity can be obtained as follows:

(10) $$N_{1}\pi {\left(\frac{d_{1}}{2}\right)}^{2}=N_{2}\pi {\left(\frac{d_{2}}{2}\right)}^{2}\Rightarrow \frac{N_{1}}{N_{2}}=\frac{d_{2}^{2}}{d_{1}^{2}}$$

where $N_{1}$ and $N_{2}\;$are the number of pores of any two different pore sizes under the same porosity. Their specific surface area ratio can be further obtained as

(11) $$\frac{a_{1}}{a_{2}}=\frac{N_{1}\pi d_{1}}{N_{2}\pi d_{2}}=\frac{d_{2}}{d_{1}}$$

where $a_{1}$ and $a_{2}$ are the specific surface areas of any two different pore sizes for the same porosity. Based on the blocking effect of friction between the pore fluid and pore wall, the permeability coefficient is assumed to be inversely proportional to the specific surface area of pores. Taking the permeability coefficient $k_{max}$ of the maximum pore size region as a reference, the permeability coefficient $k_{0i}$ of any pore size region can be defined as follows:

(12) $$k_{0i}=\chi \left(d_{i}\right)k_{max}\frac{a_{max}}{a_{i}}=\chi \left(d_{i}\right)k_{max}\frac{d_{i}}{d_{max}}$$

where $\chi (d_{i})$ is the correction coefficient considering the adsorption effect of the pore wall on the fluid, which is 0.3~0.5 for gel pores, 0.5~0.8 for capillary pores, and 1.0 for non-capillary pores. $a_{max}$ and $d_{max}$ are the specific surface area and the aperture for the maximum size of pores, respectively, and $a_{i}$ and $d_{i}$ are the specific surface area and the aperture for pores of any size, respectively. Given the pore size distribution curve and the permeability coefficient $k_{0}$ of cement mortar, the permeability coefficient of the maximum pore size region can be derived as

(13) $$\begin{array}{l} k_{0}=\int_{d_{min}}^{d_{max}}\;\chi (d_{i})k_{max}\frac{d_{i}}{d_{max}}\frac{\phi (d_{i})}{\Phi_{0}}dd_{i}\\ \Rightarrow k_{max}=k_{0}(\int_{d_{min}}^{d_{max}}\;\chi (d_{i})\frac{d_{i}}{d_{max}}\frac{\phi (d_{i})}{\Phi_{0}}dd_{i}{)}^{-1} \end{array}$$

Combined with Formula (12), the permeability coefficient of cement mortar during the freezing process can be estimated as follows:

(14) $$k=\int_{d_{min}}^{d_{cr}}\;\chi (d_{i})k_{max}\frac{d_{i}}{d_{max}}\frac{\phi (d_{i})}{\Phi_{0}}dd_{i}$$

where $\varphi (d_{i})$ is the pore size distribution function, which describes the volume percentage of pores with a diameter of $d_{i}$, and $d_{cr}$ is the critical aperture for a freezing temperature. Based on thermodynamic theory, scholars have established an expression for the critical pore size at any low temperature $T_{f}$ [51,52] as

(15) $$d_{cr}=-\frac{2\gamma }{\rho_{w}L(\ln T_{f}-\ln T_{0})}$$

where $T_{f}$ is the freezing point of pore water, $T_{0}=273.15\;K$ is the freezing point of water in the standard state, γ is the superficial tension of the ice–water interface, usually taken as $\gamma =39\times 10^{-3}\;N/m$, $\rho_{w}=1000\;kg/m^{3}$ is the mass density of water, and L=333.5 kJ/kg is the latent heat of the phase transition of water.

It was found [53] that there is a water film with a thickness of about $\delta =1.97{\left|T_{f}-T_{0}\right|}^{-\frac{1}{3}}$ adsorbed on the pore walls of concrete pores, and this water film would not freeze even at extremely low temperatures. Therefore, the critical pore diameter needs to be modified as

(16) $$d_{cr}=-\frac{2\gamma }{\rho_{w}L(\ln T_{f}-\ln T_{0})}+1.97{\left|T_{f}-T_{0}\right|}^{-\frac{1}{3}}$$

Assume that there is only water and ice in the pores, i.e., that they always remain saturated. Thus, the ice saturation at any low temperature can be obtained as

(17) $$s_{c}=\frac{\int_{d_{cr}}^{d_{max}}\;\phi (d_{i})dd_{i}}{\int_{d_{min}}^{d_{max}}\;\phi (d_{i})dd_{i}}=\frac{\int_{d_{cr}}^{d_{max}}\;\phi (d_{i})dd_{i}}{\Phi_{0}}$$

**Figure 3:**
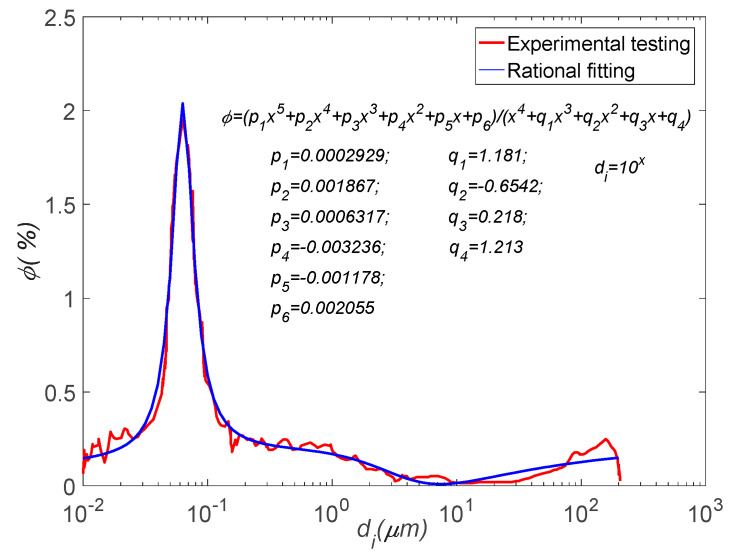
The pore size distribution curve of concrete.

**Figure 4:**
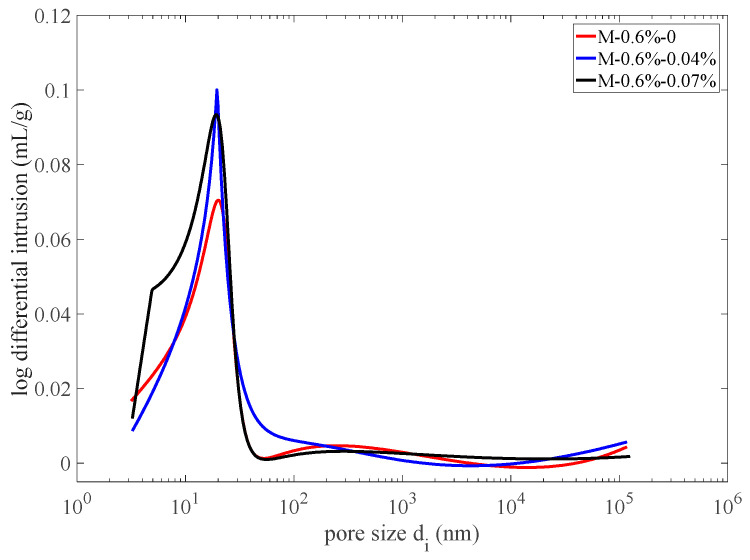
The pore size distribution fitting curves of concrete with different air-entraining contents.

Mercury intrusion porosimetry (MIP) [54] is a common method for evaluating the microstructure of porous media. This method measures the pore size and distribution by injecting mercury into the pores of the material, thereby analyzing the pore structure characteristics of the material. MIP was used to measure non-air-entrained concrete samples, and the test curve of the pore size distribution is shown in Figure 3. The rational fitting curve can be obtained as follows:

(18) $$\varphi (x)=\frac{p_{1}x^{5}+p_{2}x^{4}+p_{3}x^{3}+p_{4}x^{2}+p_{5}x+p_{6}}{x^{4}+q_{1}x^{3}+q_{2}x^{2}+q_{3}x+q_{4}}$$

where $x=\log_{10}d_{i}$, and the fitting coefficients are presented in Figure 3. To investigate the effect of air entrainment in concrete on its frost resistance, the test data on the pore size distribution of air-entrained concrete in reference [55] are adopted. That is, the same water–cement ratio of 0.38, the same water-reducing agent of 0.6%, and the corresponding pore size distribution fitting curves with no air-entraining agents and with 0.04% and 0.07% air-entraining agents added are shown in Figure 4. Their porosities are 9.36%, 12.1%, and 12.6%, respectively. It can be seen from the figure that after the air-entraining agents are added, the workability of concrete is improved. This results in a reduction in large-sized pores while resulting in a significant increase in gel pores and a slight increase in capillary pores.

**Figure 5:**
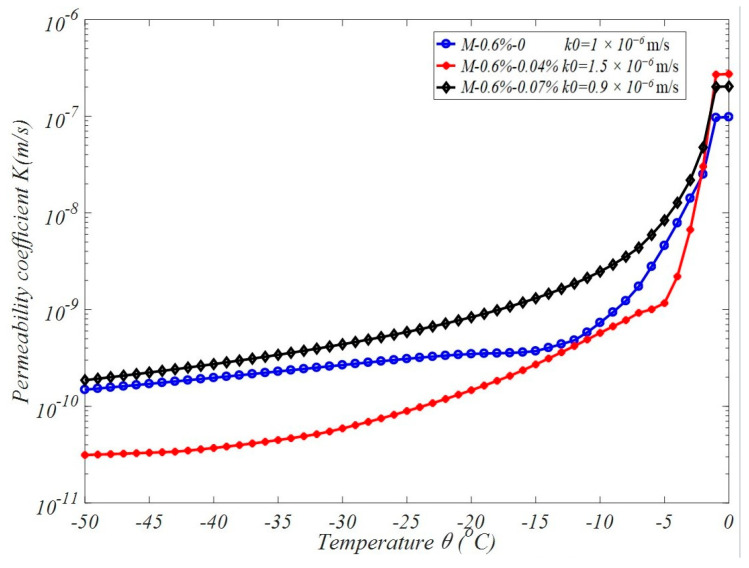
The effective permeability coefficient vs. the temperature.

Due to the complexity of the expression of the aperture distribution fitting curve, it is difficult to integrate directly, and Gaussian integration is not suitable for the entire domain because the distribution is extremely uneven. Therefore, this paper proposes to perform Gaussian integration in sections, namely, in [$d_{min}$, 0.1], [0.1, 1.0], [1.0, 10.0], and [10.0, $d_{max}$], and then accumulate these results to obtain the final integration result. Considering the characteristics of the higher-order rational curve of Equation (18), six Gaussian integration points are used in each segment for integration to achieve higher accuracy. Based on the fact that a large number of microbubbles generated by air entrainment break the connectivity of internal pores in concrete and increase their pore size, it is assumed that the permeability coefficient under standard conditions is $k_{0}=10^{-6}\;m/s$ for non-air-entrained concrete, $k_{0}=1.5\times 10^{-6}\;m/s$ for concrete with 0.04% air-entraining agents, and $k_{0}=0.9\times 10^{-6}\;m/s$ for concrete with 0.07% air-entraining agents. Therefore, the evolution trend in the permeability coefficient with decreasing temperature can be obtained from Formulas (13), (14) and (16), as shown in Figure 5. Obviously, compared to the permeability coefficient in the standard state, the permeability coefficient decreases by an order of magnitude with the decrease in temperature during the freezing process for ordinary concrete. Although the permeability coefficient of air-entrained concrete is far less than that of ordinary concrete, the permeability coefficient of air-entrained concrete is greater than that of ordinary concrete during freezing. This is because the internal gel pores and capillary pores of air-entrained concrete account for a large proportion of the pores, and the freezing point of water in these small pore holes is very low.

### 2.3. Pore Pressure

Due to the capillary surface tension, there is a pressure difference between ice and water in the pore space, which can be obtained from Laplace’s formula [56]:

(19) $$p_{c}-p_{w}=\gamma \kappa_{c}$$

where $p_{c}$ is the pore-ice pressure, $p_{w}$ is the pore-water pressure, $\kappa_{c}$ is the curvature of the ice–water contact surface at equilibrium, and γ is the superficial tension of the ice–water interface. The relationship between the adsorption-layer-water pressure $\pi_{wi}$ and pore-ice pressure is

(20) $$\pi_{wi}=p_{c}-\gamma \kappa_{\delta }$$

where $\kappa_{\delta }$ is the curvature of the contact surface between pore ice and the adsorption layer water film. In the current research, it is generally assumed that the ice–water contact surface in the pore is spherical, and the contact surface between pore ice and the adsorbed water film is cylindrical. Thus, the curvatures $\kappa_{c}$ and $\kappa_{\delta }$ are obtained as

(21) $$\kappa_{c}=\frac{4}{d_{cr}},\kappa_{\delta }=\frac{2}{d_{i}-2\delta }(d_{i}>d_{cr})$$

From Equations (19)–(21), the adsorption layer water pressure $\pi_{w}$ can be obtained as

(22) $$\pi_{wi}=p_{w}+\gamma \left(\frac{4}{d_{cr}}-\frac{2}{d_{i}-2\delta }\right)$$

Therefore, the average pore pressure $p^{*}$ of the cement mortar’s representative volume element (RVE) for any temperature θ can be expressed as

(23) $$\begin{array}{l} p^{*}(\theta )=\frac{1}{\Phi_{0}}\int_{d_{min}}^{d_{cr}}\;p_{w}(\theta )\phi (d_{i})dd_{i}+\frac{1}{\Phi_{0}}\int_{d_{cr}}^{d_{max}}\;\pi_{wi}\phi (d_{i})dd_{i}\\ =p_{w}(\theta )+\frac{2\gamma }{\Phi_{0}}\int_{d_{cr}}^{d_{max}}\;\left(\frac{2}{d_{cr}}-\frac{1}{d_{i}-2\delta }\right)\phi (d_{i})dd_{i} \end{array}$$

where θ is the temperature in Celsius, i.e., $\theta =T_{f}-T_{0}$.

Let $X(\theta )=\frac{2\gamma }{\Phi_{0}}\int_{d_{cr}}^{d_{max}}\left(\frac{2}{d_{cr}}-\frac{1}{d_{i}-2\delta }\right)\phi (d_{i})dd_{i}$. Then, the average pore pressure $p^{*}$ is rewritten as

(24) $$p^{*}\left(\theta \right)=p_{w}\left(\theta \right)+X(\theta )$$

**Figure 6:**
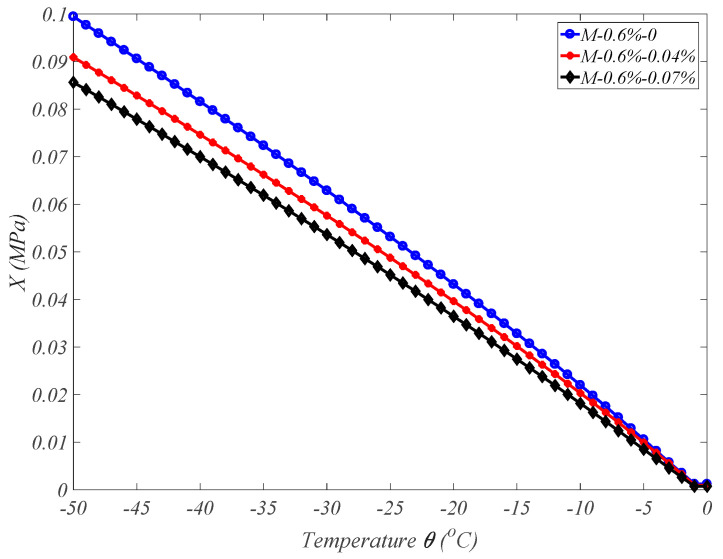
The evolution of pressure X(θ) during freezing for three types of concrete.

Similarly, the X(θ) part can be calculated by Gaussian integration in sections. For the above three types of concrete, combing Formula (16) for the critical pore diameter, the evolution of pressure from the X(θ) part with the decreasing temperature is obtained as in Figure 6. It can be seen that the pressure from the X(θ) part in air-entrained concrete is much lower than that in ordinary concrete during the freezing process because, in air-entrained concrete, the proportion of gel pores significantly increases, while non-capillary pores are not easily formed due to better workability and a more viscous slurry. Therefore, the average pore pressure in air-entrained concrete is much lower than that in ordinary concrete during the freezing process, where the osmotic pressure $p_{w}$ and temperature θ can be obtained by the numerical simulation of thermal–hydraulic–mechanical coupling fields.

## 3. Fracture Phase-Field Method

### 3.1. Brittle Fracture Theory

Francfort and Marigo [57] proposed a variational approach to the fracture problem based on Griffith’s theory. The energy required to produce each unit area of the fracture surface is equal to the critical energy release rate of that material, $G_{c}$. The total potential energy of the system $\Psi_{opt}(u,\Gamma )$ consists of three parts, i.e., the elastic strain potential energy, the dissipation potential energy due to fracture, and the potential energy of the external force, namely,

(25) $$\Psi_{opt}\left(u,\Gamma \right)=\int_{\Omega }\psi_{\varepsilon }\left(\varepsilon \right)d\Omega +\int_{\Gamma }G_{c}dS-\int_{\Omega }F\cdot ud\Omega -\int_{\partial \Omega_{hi}}f\cdot u\mathrm{dS}$$

where Γ is the crack surface, F denotes the body force per volume, f is the boundary traction, and $\psi_{\varepsilon }\left(\varepsilon \right)$ is the elastic strain energy density. For isotropic linear elastic materials, Miehe [58] and others used $\psi_{\varepsilon }(\varepsilon )=\frac{1}{2}\lambda \varepsilon_{ii}\varepsilon_{jj}+\mu \varepsilon_{ij}\varepsilon_{ij}$, where λ and μ are Lamé constants.

### 3.2. Phase-Field Approximation of Fracture Energy

Based on fracture variational theory, Bourdin et al. [36] defined a damage scalar ϕ varying in the interval [0, 1] as the fracture phase. ϕ=0 represents an intact phase, and ϕ=1.0 represents a complete fracture phase. The dissipation energy generated by cracks should be equal to the dissipation energy generated by damage, i.e.,

(26) $$\int_{\Gamma }G_{c}dS=\int_{\Omega }G_{c}\zeta d\Omega$$

where $G_{c}$ is the critical energy release rate, and ζ is the dissipation energy density, which can be defined as follows:

(27) $$\zeta (\phi ,\nabla \phi )=\frac{1}{2l_{0}}(\phi^{2}+{l_{0}}^{2}{\left|\nabla \phi \right|}^{2})$$

where $l_{0}$ is a length parameter that controls the spread of damage. Therefore, the surface energy of the crack inside the body is further expressed as

(28) $$\int_{\Gamma }G_{c}dS=\int_{\Omega }G_{c}\left[\frac{\varphi^{2}}{2l_{0}}+\frac{l_{0}}{2}|\nabla \varphi {|}^{2}\right]d\Omega$$

Assuming that the crack is generated only by the driving of tensile strain [35], the strain tensor ε is decomposed into a tension portion and a compression portion, i.e.,

(29) $$\left\{\begin{array}{c} \varepsilon_{+}=\sum_{a=1}^{3}{\left\langle \varepsilon_{a}\right\rangle }_{+}n_{a}⊗n_{a}\\ \varepsilon_{-}=\sum_{a=1}^{3}{\left\langle \varepsilon_{a}\right\rangle }_{-}n_{a}⊗n_{a} \end{array}\right.$$

where $\varepsilon_{+}$ and $\varepsilon_{-}$ are tensile and compressive strain tensors, respectively, $\varepsilon_{a}$ is the principal strain, $n_{a}$ is the principal direction vector, and the operators ${\left\langle \cdot \right\rangle }_{+}$ and ${\left\langle \cdot \right\rangle }_{-}$ are defined as $\langle \cdot \rangle_{+}=(\cdot +|\cdot |)/2$, $\langle \cdot \rangle_{-}=(\cdot -|\cdot |)/2$. Substituting the decomposed strain tensor into the elastic energy density equation, the strain energy density in tension and compression are obtained as follows, respectively:

(30) $$\left\{\begin{array}{c} \psi_{\varepsilon }^{+}(\varepsilon )=\frac{\lambda }{2}{\left\langle tr\left(\varepsilon \right)\right\rangle }_{+}^{2}+\mu tr\left(\varepsilon_{+}^{2}\right)\\ \psi_{\varepsilon }^{-}(\varepsilon )=\frac{\lambda }{2}{\left\langle tr\left(\varepsilon \right)\right\rangle }_{-}^{2}+\mu tr\left(\varepsilon_{-}^{2}\right) \end{array}\right.$$

Borden [59] assumed that only the tensile strain energy portion is discounted due to fracture, and thus, the elastic strain energy density is expressed as

(31) $$\psi_{\varepsilon }(\varepsilon )=\left[\left(1-m\right){\left(1-\varphi \right)}^{2}+m\right]\psi_{\varepsilon }^{+}(\varepsilon )+\psi_{\varepsilon }^{-}(\varepsilon )$$

where m is a parameter much smaller than one and introduced for numerical convergence.

### 3.3. Governing Equation

The total Lagrangian energy functional can be expressed as the sum of the elastic potential energy (29), the fracture energy (26), and the external potential energy due to the external loads:

(32) $$\begin{array}{ll} L & =\int_{\Omega }\;\left\{\left[\left(1-m\right){\left(1-\varphi \right)}^{2}+m\right]\psi_{\varepsilon }^{+}\left(\varepsilon \right)+\psi_{\varepsilon }^{-}\left(\varepsilon \right)\right\}d\Omega \\ & +\int_{\Omega }\;G_{c}\left[\frac{\varphi^{2}}{2l_{0}}+\frac{l_{0}}{2}|\nabla \varphi {|}^{2}\right]d\Omega -(\int_{\Omega }\;F\cdot ud\Omega +\int_{\partial \Omega_{hi}}\;f\cdot udS) \end{array}$$

Disregarding the kinetic energy term, based on the fracture dissipation energy and elastic strain energy, the strong form of the fracture phase-field governing equation is obtained according to the variational principle as follows:

(33) $$\begin{array}{c} \nabla \cdot \sigma +F=0\\ \left[\frac{2l_{0}\left(1-m\right)\psi_{\varepsilon }^{+}}{G_{c}}+1\right]\varphi -\frac{2l_{0}\left(1-m\right)\psi_{\varepsilon }^{+}}{G_{c}}=l_{0}^{2}\nabla^{2}\varphi \end{array}$$

where σ is the Cauchy stress tensor, $\sigma =\left[\left(1-m\right){\left(1-\varphi \right)}^{2}+m\right]\frac{\partial \psi_{\varepsilon }^{+}}{\partial \varepsilon }+\frac{\partial \psi_{\varepsilon }^{-}}{\partial \varepsilon }$.

According to the second law of thermodynamics, crack formation is an irreversible process. Thus, a maximum historical tensile strain energy density field, $H\left(x,t\right)=\underset{s\in \left[0,t\right]}{max}\psi_{e}^{+}\left(\varepsilon \left(x,t\right)\right)$, is introduced to replace the tensile strain energy density field. The weak forms of the fracture phase field can be obtained as

(34) $$\begin{array}{c} \int_{\Omega }\sigma :\delta \varepsilon \mathrm{dV}-\left(\int_{\Omega }F\cdot \delta u\mathrm{dV}+\int_{\partial \Omega_{hi}}f\cdot \delta u\mathrm{dS}\right)=0\\ \int_{\Omega }\left\{\left[\frac{2l_{0}\left(1-m\right)H}{G_{c}}+1\right]\varphi \delta \varphi -\frac{2l_{0}\left(1-m\right)H}{G_{c}}\right\}\mathrm{dV}=\int_{\Omega }l_{0}^{2}\nabla \varphi \cdot \nabla \delta \varphi \mathrm{dV} \end{array}$$

By discretizing the above weak-form integral equations within the finite element framework and adopting the staggered solving scheme, the whole fracture process can be simulated.

## 4. Thermal–Hydraulic–Mechanical Coupling Model for Frost Damage Process in Concrete

Frost damage in concrete is the result of the coupling of the stress field, seepage field, and temperature field in a porous system. The governing equations of the three fields are discussed below.

### 4.1. Stress Field

For porous systems [60],

(35) $$\sigma =\sigma^{\prime }-bp^{*}I$$

where σ is Cauchy stress, $\sigma^{\prime }$ is effective stress, $p^{*}$ is average pore pressure, I is a unit matrix, b is Biot’s ratio, and $b=1-K_{0}/K_{m},\;$with $K_{0}$ and $K_{m}$ being the bulk elastic moduli of porous systems and skeletons.

The effective stress is related to the strain as follows:

(36) $$\sigma^{\prime }=D\varepsilon^{e}=D(\varepsilon -\varepsilon_{th}I)=D\left(\varepsilon -\alpha^{L}(\theta -\theta_{ref})I\right)$$

where D is the stiffness matrix, ε is the total strain, $\varepsilon_{th}$ is the thermal strain due to temperature differences, $\alpha^{L}\left(1{/}^{o}C\right)$ is the coefficient of linear expansion, $\theta \left({}^{\circ}C\right)$ is the temperature, and $\theta_{ref}\left({}^{\circ}C\right)$ is the reference temperature of the thermal strain. The mechanical equilibrium differential equation is

(37) ∇⋅σ+F=0

where ***F*** is body force. Then, in the absence of external loads,

(38) $$\left\{\begin{array}{c} \frac{\partial {\sigma^{\prime }}_{x}}{\partial x}+\frac{\partial \tau_{yx}}{\partial y}+\frac{\partial \tau_{zx}}{\partial z}-b\frac{\partial p^{*}}{\partial x}=0\\ \frac{\partial \tau_{xy}}{\partial x}+\frac{\partial {\sigma^{\prime }}_{y}}{\partial y}+\frac{\partial \tau_{zy}}{\partial z}-b\frac{\partial p^{*}}{\partial y}=0\\ \frac{\partial \tau_{xz}}{\partial x}+\frac{\partial \tau_{yz}}{\partial y}+\frac{\partial {\sigma^{\prime }}_{z}}{\partial z}-b\frac{\partial p^{*}}{\partial z}=0 \end{array}\right.$$

### 4.2. Seepage Flow Field in Porous Systems

From the conservation of mass, the constitutive equations of the phases, and Darcy’s law for water migration in porous systems, it can be deduced that

(39) $$\beta {\dot{p}}_{w}=\nabla \cdot \left(\frac{k}{\eta }\nabla p_{w}\right)+S-b{\dot{\varepsilon }}_{v}$$

where $p_{w}$ is the osmotic pressure; k is the effective permeability coefficient, which can be estimated by the derived Formula (14), reflecting the variation with the freezing process when no damage occurs; η is the dynamic viscosity coefficient of water; $\varepsilon_{v}$ is the bulk strain; b is Biot’s ratio; and β is the effective compressive flexibility of porous material, which is defined as

(40) $$\beta =\frac{\Phi s_{w}}{K_{w}}+\frac{\Phi s_{i}}{K_{i}}+\frac{b-\Phi }{K_{m}}$$

where $K_{w}$, $K_{i}$, and $K_{m}$ are the bulk moduli of pore water, pore ice, and porous skeletons, respectively. $s_{w}$ and $s_{i}$ are the saturation of water and ice, respectively. Φ is the porosity of a porous system. S in Equation (39) is the source or sink term and is obtained as follows:

(41) $$S=\left(\frac{1}{\rho_{i}}-\frac{1}{\rho_{w}}\right){\dot{\varpi }}_{w\to i}+\bar{\alpha }\dot{T}-\frac{b-\Phi }{K_{m}}\dot{X}-\frac{\Phi s_{i}}{K_{i}}\dot{\kappa }$$

where $\rho_{i}$ and $\rho_{w}$ are the densities of ice and water, respectively. $\bar{\alpha }$ is the effective volume thermal expansion coefficient, which is estimated by the following expression:

(42) $$\bar{\alpha }=\Phi s_{w}\alpha_{w}+\Phi s_{i}\alpha_{i}(b-\Phi )\alpha_{0}$$

where $\alpha_{w}$, $\alpha_{i}$, and $\alpha_{0}$ are the coefficients of volume thermal expansion of water, ice, and porous skeletons, and $\dot{\kappa }$ is the curvature variation of pores.

When a material is damaged, the permeability coefficient undergoes fundamental changes. According to the fact that phase-field damage is a loss of material continuity, the permeability coefficient of the porous material switches as follows:

(43) $$k=k_{0}(1+\frac{\phi }{\Phi_{0}})$$

where ϕ is the phase-field damage, $\Phi_{0}$ is the initial porosity, and $k_{0}$ is the permeability coefficient before freezing.

### 4.3. Temperature Field

For a porous media mixture, the heat transfer equation considering the latent heat of the phase change from water to ice is [15,50]

(44) $$\rho C\frac{\partial T}{\partial t}=\nabla \cdot ({\bar{\lambda }}_{m}\nabla T)+Q\frac{\partial w_{i}}{\partial t}$$

where ${\bar{\lambda }}_{m}$ is the effective thermal conductivity of the porous system, which can be estimated by Equation (9); C is the specific heat capacity of the porous system; t is time; ρ is the mass density of the system; and Q is the latent heat of the phase transition of water.

## 5. Simulation of Frost Damage in Concrete Specimens

### 5.1. Frost Damage in Ordinary Concrete Specimens

**Figure 7:**
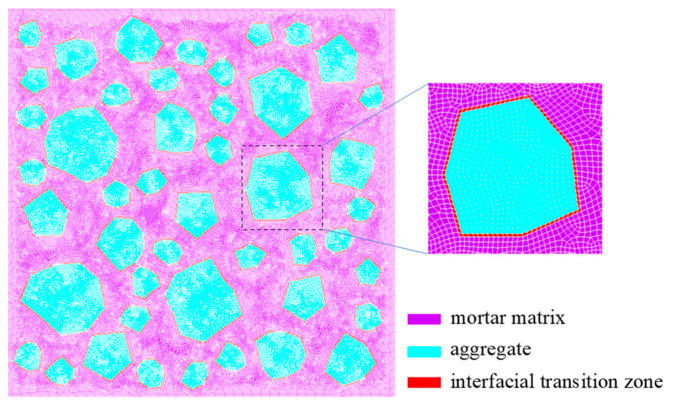
The FEM mesh of a concrete sample with randomly distributed aggregates.

A concrete specimen with a cross-section of 150 mm×150 mm was used in the simulation; it is regarded as a composite material consisting of aggregates, a cement paste, and an interfacial transition zone (ITZ). The randomly distributed aggregates are graded with three grades, and the thickness of the ITZ is set to 0.3~0.6 mm, as shown in Figure 7. The initial temperature field is assumed to be uniformly distributed with a value of 275.15 K. The temperature load is applied to the surrounding boundary. The finite element model of the concrete sample at the mesoscopic scale is presented in Figure 7. It is assumed that the aggregates in concrete never fail. The material parameters of the components in concrete are given in Table 1 [61].

**Figure 8:**
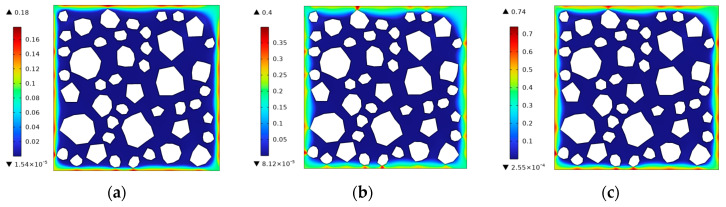
The frost damage distribution during freezing. (**a**) θ=0 °C, *t* = 2040 s. (**b**) θ=-5 °C, *t* = 3840 s. (**c**) θ=-10 °C, *t* = 5640 s.

The fracture phase-field method was used to simulate the freezing and fracturing process of this concrete specimen. The frost cracking results obtained when the air entrainment of concrete is 0% and the cooling rate is 10 °C/h are shown in Figure 8. It can be seen that the frost damage begins to appear near the sharp edges of the aggregate at the specimen boundary when the boundary temperature drops to 0  °C. As the temperature continues to decrease, the boundary damage further develops into cracks, extending into the interior of the concrete and toward the ITZ of the aggregate.

### 5.2. Frost Damage in Concrete with Different Microbubble Contents

Adding air-entraining agents will produce many microbubbles in the cement mortar; these microbubbles have low-temperature pumping, a liquid storage tank effect, and a buffering effect on frost expansion. Thus, the average pore pressure Formula (24) is further modified as follows:

(45) $$p^{*}=(1-c)(p_{w}+X(\theta ))$$

where *c* is the microbubble content.

**Figure 9:**
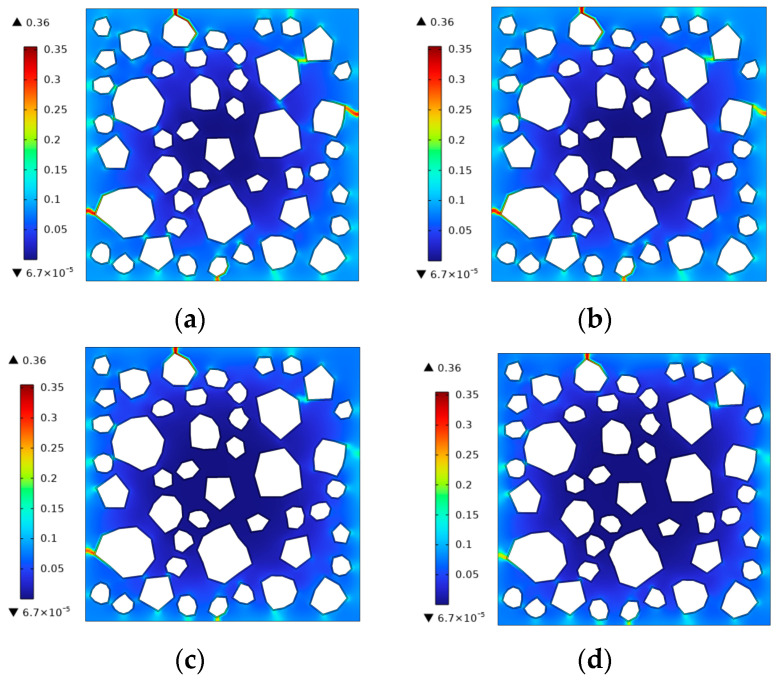
The frost damage distribution in concrete with different microbubble contents at a freezing temperature θ=−10 °C. (**a**) c = 3%. (**b**) c = 6%. (**c**) c = 9%. (**d**) c = 10%.

In this study, the frost damage simulation was carried out for concrete with microbubble contents of c=3%,6%,9%, and 10%. The frost damage results of the calculations are shown in Figure 9 at a temperature of θ=−10 °C. It can be seen that as the content of microbubbles increases, the degree of damage to the concrete surface gradually decreases. When the microbubble content is 10%, the damage to concrete is minimized, which is also consistent with experimental results [62].

### 5.3. Frost Damage in Concrete with Different Cooling Rates

**Figure 10:**
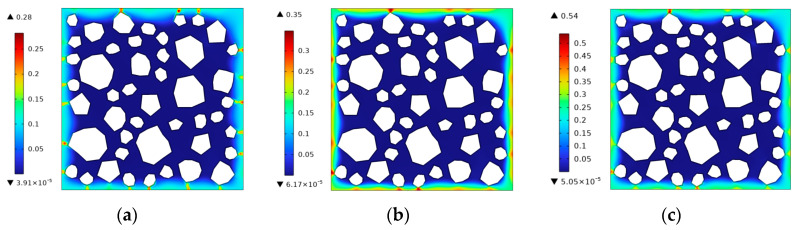
The frost damage distribution at different cooling rates when the temperature drops to −10 °C. (**a**) $\dot{\theta }=5\;{}^{\circ}C/h$. (**b**) $\dot{\theta }=10\;{}^{\circ}C/h$. (**c**) $\dot{\theta }=20\;{}^{\circ}C/h$.

The cooling rate has a great impact on the degree of frost damage to concrete. Taking the concrete specimen containing 10% microbubbles as an example, the frost damage process in concrete with cooling rates of $\dot{\theta }=5\;{}^{\circ}C/h,10\;{}^{\circ}C/h,20\;{}^{\circ}C/h$ was simulated. The frost damage results when the temperature drops to −10 °C are shown in Figure 10. It is easy to see from the damage comparisons that the faster the cooling, the more severe the frost damage.

**Figure 11:**
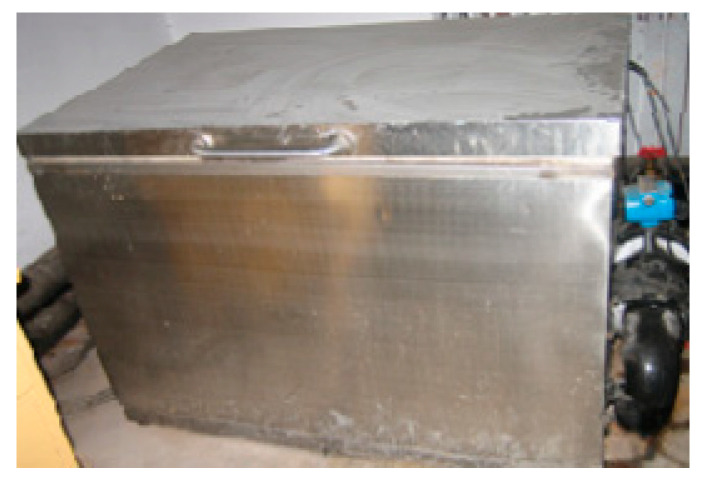
Fully automatic rapid freeze–thaw testing machine.

**Figure 12:**
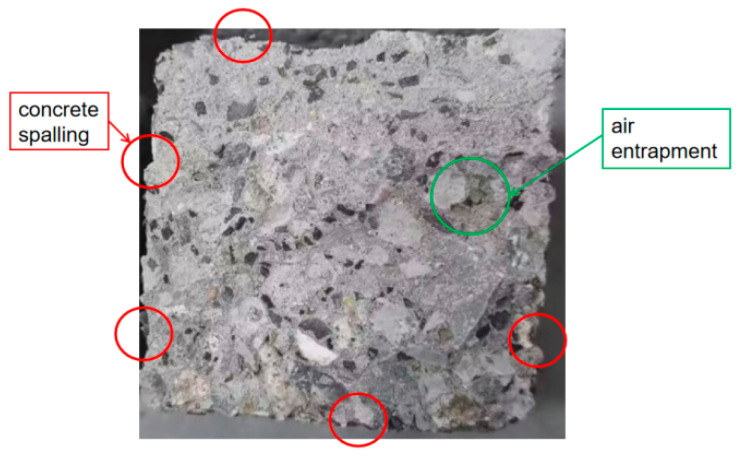
Damage to concrete specimens in freeze–thaw experiments.

The freeze–thaw cycle test was conducted using a fully automatic rapid freeze–thaw testing machine for concrete (Figure 11). Each freeze–thaw cycle was completed within 2–04 h. The damage to the concrete specimen after 200 freeze–thaw cycles is shown in the image in Figure 12. The edge of the concrete specimen peels off, and the defects caused by internal bubbles are also exposed. The concrete specimen edge’s freeze–thaw damage pattern is similar to that in the frost damage simulation.

## 6. Conclusions

This article presents mesoscopic numerical simulation research on the frost-cracking process in concrete. Considering that the internal pores and microbubbles generated by air entrainment in concrete are relatively small in size compared to aggregates, it is difficult to directly represent them in mesoscopic numerical models. Therefore, this article first focuses on the homogenization of the effective thermal conductivity and permeability coefficients of the mortar in concrete, reflecting the ice-crystal phase transformation mechanism of pore water, with the aim being to explore the evolution mechanism of thermal conductivity and permeability during freezing. Secondly, based on the fracture phase-field method and the established estimation models of the thermal conductivity and permeability coefficient for a porous mixture, a finite element numerical simulation of thermal–hydraulic–mechanical coupling at the mesoscale was carried out to explore the frost fracture mode of concrete during the freezing process and the influence of microbubbles and the cooling rate on the frost cracking of concrete. Finally, some conclusions are summarized below.

Based on the ice-crystal phase transition mechanism of pore water and the theory of microscopic inclusions, an estimation model for macroscopic thermal conductivity and permeability coefficients that can reflect the characteristics of the pore size distribution is established, where the integration calculation involving the pore size distribution curve is carried out by a segmented Gaussian integration strategy.

A permeability coefficient switching model considering the fundamental changes in permeability caused by frost cracking is proposed based on the notion that the fracture phase is a loss of continuity.

According to the proposed estimation model, evolution laws reflecting that heat conduction continuously increases while the permeability coefficient decreases in a cliff-like manner are obtained. The weakening mechanism of microbubbles on the heat conduction coefficient is also discovered. In addition, although the permeability of air-entrained concrete decreases continuously during the cooling process, the degree of change is relatively small compared to ordinary concrete. This is because small pore sizes account for a large proportion of pores in air-entrained concrete, and there are few non-capillary pores.

Based on the high-quality pore size distribution curve of air-entrained concrete, it is concluded that the average pore pressure of air-entrained concrete during the freezing process is much lower than that of ordinary concrete. Regarding the storage and cold suction effect of microbubbles, the average pore pressure is reduced according to the content of microbubbles, and the mechanism of frost resistance of air-entrained concrete is demonstrated.

Based on the established thermal–hydraulic–mechanical coupling finite element model of concrete at the mesoscale, a frost damage simulation of the freezing process was successfully carried out for ordinary concrete and air-entrained concrete with different microbubble contents. The results show that the cracks first occurred on the concrete sample surface near the aggregate and then extended inward along the interface transition zone, which is consistent with the frost-cracking scenario of concrete structures in cold regions. At the same cooling rate and temperature, the phase-field damage in non-air-entrained concrete is 0.74, while the maximum phase-field damage in air-entrained concrete is 0.35. This demonstrates the frost resistance effect of incorporating air-entraining agents into the concrete. For two concrete specimens with cooling rates of 20 °C/h and 5 °C/h, when the temperature is lowered to the same level, the phase-field damage to the concrete specimen with a cooling rate of 20 °C/h is 0.54, while the maximum phase-field damage of the concrete specimen with a cooling rate of 5 °C/h is 0.28. An adverse effect of accelerated cooling on frost cracking is also discovered.

## Figures and Tables

**Table 1:** Concrete parameters for fracture phase-field modeling.

Component in Concrete	Density ρ (kg/m^3^)	Elastic Modulus E (GPa)	Poisson’s Ratio μ	Length Scale l0 (mm)	Critical Energy Release Rate Gc (J/m^2^)
Aggregate	2600	70.0	0.2		
Mortar matrix	2140	10.1	0.2	0.45	15.0
ITZ	2140	7.0	0.2	0.3	10.0

## Data Availability

The original contributions presented in the study are included in the article, further inquiries can be directed to the corresponding author.
